# Bariatric Surgery and Type 2 Diabetes Mellitus: Assessing Factors Leading to Remission. A Systematic Review

**DOI:** 10.7759/cureus.9973

**Published:** 2020-08-23

**Authors:** Shahbakht Ilyas, Reham Al-Refai, Reeju Maharjan, Liliana Diaz Bustamante, Kyrillos N Ghattas, Safeera Khan

**Affiliations:** 1 Surgery, California Institute of Behavioral Neurosciences and Psychology, Fairfield, USA; 2 Medicine and Surgery, CMH Lahore Medical College and Institute of Dentistry, Lahore, PAK; 3 Pathology, California Institute of Behavioral Neurosciences and Psychology, Fairfield, USA; 4 Neurology, California Institute of Behavioral Neurosciences and Psychology, Fairfield, USA; 5 Family Medicine, California Institute of Behavioral Neurosciences and Psychology, Fairfield, USA; 6 Internal Medicine, California Institute of Behavioral Neurosciences and Psychology, Fairfield, USA; 7 Internal Medicine, Assiut University, Assiut, EGY

**Keywords:** bariatric surgery, type 2 diabetes mellitus, remission, diabetes

## Abstract

Type 2 Diabetes Mellitus (T2DM) is a health problem of paramount proportions and is associated with significant morbidity and mortality. Our study aims to review data published on the effects of different types of bariatric surgeries on T2DM remission, compared to lifestyle and medical intervention (LMI) exclusively, along with a comprehensive finding of numerous preoperative factors that lead to remission. We used PubMed, PubMed Central (PMC), and MEDLINE to search for literature. Our criteria included peer-reviewed, English language articles published in 2010 and onwards, consisting of adults with T2DM and a body mass index (BMI) of >30 kg/m^2^ as the population of interest. Twenty-four articles with 5,411 patients were selected for this systematic review, which included nine randomized controlled trials (RCTs) and 15 observational studies. The primary endpoint was T2DM remission. Based on the review, bariatric surgery is superior to LMI in inducing remission in T2DM, especially when employing the Roux-en-Y Gastric Bypass (RYGB) technique. Lower age of onset and shorter duration of T2DM, along with a high BMI are some of the factors that can lead to greater remission rates. Further research in RCTs is needed by incorporating double/triple-blind protocols, a standard definition of T2DM remission, long follow-up periods to evaluate for relapses in remission and any side effects, with a focus on inflammatory markers (eg, osteopontin), scoring systems (eg, DiaRem), and benefits of One-Anastomosis Gastric Bypass (OAGB) over other modalities, to advance our understanding of T2DM remission.

## Introduction and background

Obesity is one of the leading global causes of preventable deaths and has rapidly become a health issue of epidemic proportions, impacting people of all ages, from children and adolescents to adults [1,2,3]. The connection between obesity and diabetes has been well researched and documented [4]. In the preceding decade, there has been a significant rise in the incidence of Type 2 Diabetes Mellitus (T2DM), with numbers expected to reach 439 million by the year 2030 [5]. 

The pathophysiology of T2DM consists of impaired insulin production by the pancreas and the ineffective use of insulin (insulin resistance) in the liver and periphery [6]. Ninety percent of all T2DM patients have excess body weight, which is associated with an increased likelihood of developing complications such as hypertension and cardiovascular disease [4]. The risk of developing T2DM is directly related to an individual's body mass index (BMI). This risk can range from 2%, for BMI 25 to 29.9 kg/m^2^, to 13%, for BMI >35 kg/m^2^ [7]. Management of T2DM includes weight loss and pharmacotherapy. However, many oral hypoglycemic agents and insulin therapy cause further weight gain as an adverse effect [8]. The desired goals for the treatment are hemoglobin A1c (HbA1c) <7.0%, low-density lipoprotein (LDL) cholesterol <100 mg/dL, and systolic blood pressure <130 mmHg [9]. Attaining these goals for preventing long-term complications, however, is unsuccessful in as many as 90% of T2DM patients [10]. 

Bariatric surgery was originally intended for weight reduction only but was added later as a treatment option by the American Diabetes Association (ADA) for the management of obese diabetics in 2009 [11]. By 2011, it was recognized by the International Diabetes Foundation (IDF) within their position statement as a treatment option for T2DM and obesity [12]. The general criteria for bariatric surgery is obese patients with a BMI >40 kg/m^2^ or >35 kg/m^2^ with obesity-related comorbidities [13]. Various types of bariatric surgical techniques have been studied and are used for weight reduction and treatment of comorbidities. These include Gastric Banding (GB), Roux-en-Y Gastric Bypass (RYGB), Biliopancreatic Diversion (BPD), and Sleeve Gastrectomy (SG) [14]. 

Numerous studies have shown that bariatric surgery improves glycemic control in T2DM, with many individuals even showing remission [15]. Randomized controlled trials (RCTs) have compared the effectiveness of bariatric surgery against traditional T2DM management, with results indicating a higher likelihood of T2DM remission after surgery, as opposed to lifestyle and medical intervention (LMI) [16]. There is also evidence that bariatric surgery decreases the number of deaths from diabetes, with up to 92% decrease in mortality in patients with T2DM [17]. 

A problem with data available for bariatric surgery, for resolution of T2DM, is the lack of sufficient long-term follow-up studies for better assessment of remission rates and to check for relapse [18]. Another shortcoming is the insufficient availability of RCTs that are free from inherent bias, and adopt double- or triple-blind protocols. In this systematic review, we aim to gather information on bariatric surgery, and its role in inducing remission in T2DM, while drawing inference on the numerous factors that lead to this remission. These factors include preoperative predictors, comparison of different types of bariatric surgeries, and the difference in follow-up times and how they affect remission rates.

## Review

Methods

Search Strategy

**Figure 1:**
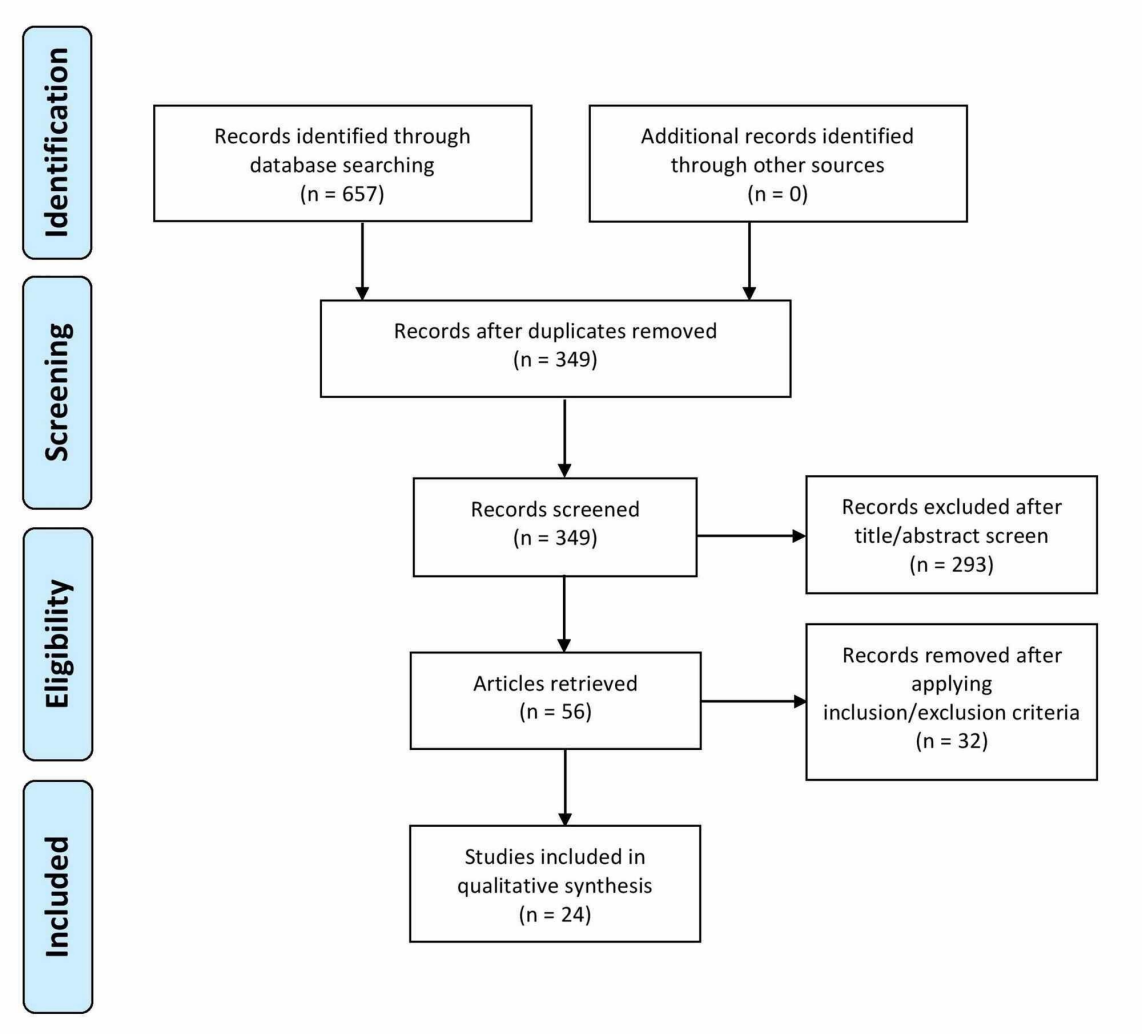
PRISMA Flow Diagram PRISMA: Preferred Reporting Items for Systematic Reviews and Meta-Analyses [19].

This systematic review was conducted using the guidelines of 2009 Preferred Reporting Items for Systematic Reviews and Meta-Analyses (PRISMA) [19]. We performed a comprehensive search of published literature in PubMed, PubMed Central (PMC), and MEDLINE. The keywords used to search the literature within these databases were as follows: “Bariatric Surgery”, “Type 2 Diabetes Mellitus”, “Remission”, and “Diabetes”. These keywords were applied separately and/or in conjunction with one another (Table 1). A total of 349 articles were selected after the removal of duplicates. We screened through each of those articles based on title and then by reviewing the abstracts. Next, we read through the full texts, applied the inclusion and exclusion criteria, and subsequently, we were left with 24 articles that we included in our review (Figure 1). 

**Table 1:** Keyword Search Results MeSH: Medical Subject Headings Filters Applied: Clinical Trial, Meta-Analysis, Observational Study, Randomized Controlled Trial, Systematic Reviews, in the last 10 years, English, Middle Aged: 45-64 years

Keywords	Regular / MeSH	Database Used	Number of Search Results
Bariatric Surgery	Regular	PubMed	1,368
Type 2 Diabetes Mellitus	Regular	PubMed	9,228
Type 2 Diabetes Mellitus and Bariatric Surgery and Remission	Regular	PubMed	110
Bariatric Surgery and Remission	Regular	PubMed	357
Diabetes and Remission	Regular	PubMed	252
Diabetes Mellitus, Type 2/Surgery and Bariatric Surgery	MeSH	PubMed	143

 

Inclusion and Exclusion Criteria

We pursued only those studies that were related to our topic of remission in T2DM post-bariatric surgery. For this systematic review, we selected only peer-reviewed, English language articles. Our search criteria included literature published in 2010 and onwards. The desired study designs included systematic reviews and meta-analysis, RCTs, and observational studies. The demographic targeted within the researched literature included adults with T2DM who had a BMI >30kg/m^2^. Exclusion criteria applied towards our search included individuals with Type 1 Diabetes Mellitus, pre-diabetes, BMI <30kg/m^2^, pediatric population, and studies with follow-up of less than a year. In addition, grey literature, case reports, comments, and animal studies were not included. 

Results

Upon reviewing the articles for suitability, we included 24 studies in our paper. Nine of these are RCTs and 15 are observational studies. A thorough quality assessment was performed by using the Cochrane Risk of Bias Tool for RCTs and The Newcastle-Ottawa Scale for observational studies. All of the studies satisfied the quality check. Six RCTs and one observational study compared bariatric surgery with medical and lifestyle interventions. Two RCTs and nine observational studies compared different types of bariatric surgeries. One RCT compared RYGB with usual diabetes care and exenatide in patients with T2DM and hypertension. Moreover, three observational studies focused on various pre-operative remission predictors. One observational study researched the role of osteopontin, and one studied the efficacy of the DiaRem score as a predictor of remission of T2DM after bariatric surgery. The primary endpoint in most of the studies was remission in T2DM. The total number of patients for our review is 5,411 (Table 2). 

**Table 2:** Summary of reviewed articles T2DM: Type 2 Diabetes Mellitus, RYGB: Roux-en-Y Gastric Bypass, HbA1c: hemoglobin A1c, BPD: Biliopancreatic Diversion, LMI: lifestyle and medical intervention, BMI: body mass index, SG: Sleeve Gastrectomy, GB: Gastric Banding, RCT: randomized controlled trial, OAGB: One-Anastomosis Gastric Bypass, HDL: high-density lipoprotein

Authors & Year of Publication	Study Type	Intervention	No. of Patients	Results	Conclusions	Follow-up Period (years)
Honarmand et al. [1]. 2017	Observational Study	RYGB	900	T2DM remission at one-year follow-up was seen in 37% of participants. The four predictive variables of remission were age, HbA1c, insulin use, and the use of any hypoglycemic drugs. Also, a DiaRem score of <5 showed greater remission. Overall, this scoring system had a moderate predictive value	DiaRem score is helpful for clinicians to determine which patients will benefit from surgery	1
Nedelcu et al. [7]. 2017	Observational Study	SG	46	All of the patients who had sustained remission at five-year follow-up (9/46) were not on insulin preoperatively. Thirty patients had improvement of T2DM at one year, and for 27, this improvement persisted at five-year follow-up	A moderate benefit was seen in achieving remission in T2DM after SG. Pre-op need for insulin, high HbA1C, and advanced age are bad prognostic factors for remission	5
Mingrone et al. [8]. 2012	RCT	LMI / RYGB / BPD	56	At two-year follow-up, T2DM remission was seen in 75% of RYGB participants and 95% of BPD participants, and 0% in the LMI group	T2DM remission is resulted after bariatric surgery, compared to LMI. Preoperative BMI levels and amount of weight loss did not prove to be predictors of improvement in T2DM	2
Wazir et al. [14]. 2019	Observational Study	GB / SG / RYGB	121	At the two-year follow-up period, the T2DM remission rate for surgery patients was 68.6%	T2DM remission was successfully achieved in patients undergoing bariatric surgery	2
Hofsø et al. [15]. 2019	RCT	RYGB / SG	107	Higher remission rates were seen in the RYGB group than SG. After RYGB, early complications were seen in 10/54 patients, while 17/53 had late complications. After SG, 8/55 had early, while 22/54 had late adverse effects	At one-year follow-up, RYGB was better than SG at achieving remission in T2DM. The effects on β-cell function were similar after both types of surgeries	1
Cummings et al. [16]. 2016	RCT	RYGB / LMI	32	Sixty percent of RYGB and 5.9% of LMI participants remitted at one-year follow-up. Medication usage was reduced after RYGB, as opposed to LMI	RYGB showed greater remission of T2DM when compared to LMI	1
Ruiz‑Tovar et al. [20]. 2019	RCT	OAGB / SG / RYGB	546	At five-year follow-up, OAGB was superior to RYGB and SG in remitting T2DM, hypertension, and dyslipidemia. At one, two, and five years, the BMI lost with OAGB was also higher than with RYGB and SG. There was no major superiority of RYGB over SG when comparing T2DM remission rates of the two	OAGB proves to be superior to RYGB and SG in achieving T2DM remission	5
Ikramuddin et al. [21]. 2016	RCT	RYGB / LMI	119	T2DM remission (complete/partial) at three years was seen in 17%/19% for RYGB, and 0% for LMI participants. More medication postoperatively was used by LMI group than RYGB group	At three-year follow-up, RYGB is associated with greater remission rates of T2DM than LMI	3
Courcoulas et al. [22]. 2015	RCT	RYGB / GB / LMI	52	T2DM remission was achieved by 40% of RYGB, 29% of GB, and none of the LMI participants. No antidiabetic medication was used by 65% of RYGB and 33% of GB at three years. However, all of the LMI participants still needed medication	Bariatric surgery resulted in more T2DM remission, compared to LMI alone	3
Mingrone et al. [23]. 2015	RCT	RYGB / BPD / LMI	53	T2DM remission at five years was seen in 50% of the surgery participants, with none seen for LMI participants. Relapse was seen in 53% of RYGB and 37% of BPD participants	Bariatric surgery is superior to LMI in achieving remission in T2DM patients	5
Courcoulas et al. [24]. 2014	RCT	RYGB / GB / LMI	50	Partial remission of T2DM was seen in 50% of RYGB, 27% of GB, and 0% of the LMI group. Complete remission, however, was achieved by 17% of RYGB, 23% of GB, and 0% of LMI participants. In both groups, there was a reduction in medication usage	At one year, RYGB proved to be better than GB for inducing remission in T2DM patients	1
Liang et al. [25]. 2013	RCT	RYGB / LMI / LMI + Exenatide	101	T2DM remission occurred in 90% of all RYGB participants, with a decline in the need for antihypertensive drugs. For LMI participants, remission was not seen	RYGB proves to be a significant factor for reduction in cardiovascular disease in T2DM patients	1
Madsen et al. [26]. 2019	Observational Study	RYGB	1111	T2DM remission was seen in 74% of RYGB participants; however, 27% of these relapsed after the five-year follow-up. Baseline predictors of non-remission include: age >50 years, HbA1c >7%, duration of diabetes >5 years, and use of anti-diabetic medications besides metformin	At one year postoperatively, RYGB showed remission in T2DM patients	5
Carbone et al. [27]. 2019	Observational Study	BPD / RYGB	41	Remission occurred in T2DM patients who had a larger waist circumference, body weight, BMI, and increased serum osteopontin levels at three years follow-up	Serum level of osteopontin prior to surgery is a useful predictor of remission at three years	3
Fernández-Soto et al. [28]. 2017	Observational Study	BPD / RYGB / SG	49	Remission was seen in >80% of T2DM patients. One year after the intervention, remission predictors included BMI levels, HbA1c levels, and insulin intake	In morbidly obese patients, there was no significant difference in remission rates after different types of bariatric surgery modalities	1
Adams et al. [29]. 2017	Observational Study	RYGB	88	T2DM remission at two-years follow-up is 75%, 62% at six years, and 51% at 12 years	RYGB is an effective method of achieving remission in T2DM, along with long-term weight loss and prevention of hypertension and dyslipidemia	12
Ghio et al. [30]. 2017	Observational Study	RYGB / SG	74	Out of the 27% of participants that observed T2DM remission at a median of 11.7 months, 65% relapsed at a median of 26.7 months. Initially, a great reduction in HbA1c levels and insulin use was seen. But these progressively declined	Preoperative insulin therapy resulted in low T2DM remission probability after bariatric surgery	4.9 + 1.9
Purnell et al. [31]. 2016	Observational Study	RYGB / GB	606	A total of 68.7% of T2DM patients who underwent RYGB and 30.2% of GB candidates were in remission at the three-year follow-up. The predictive factor for remission with both surgeries was low HbA1c without the need for insulin therapy. For GB it was lower weight, while for RYGB they were higher fasting C peptide level and lower leptin-to-fat mass ratio	Higher postoperative weight loss correlated with greater remission in T2DM, nevertheless, there was still a twofold increase rate of remission after RYGB after checking for differences in the amount of weight that is lost	3
Yska et al. [32]. 2015	Observational Study	GB / RYGB / SG / Other/ Unknown	569	An 18-fold increased chance of remission in T2DM was seen after bariatric surgery compared to control patients. At two-year follow-up, RYGB had the best result, followed by SG and GB. Other parameters like BMI, plasma glucose, triglycerides, and HbA1c were also decreased	RYGB and SG are better than GB at achieving remission	2
Bhasker et al. [33]. 2015	Observational Study	RYGB	106	Remission rates at <5 years were 100%. HbA1c decreased from 8.7±2.1 to 6.2± 1.3%. Predictors of remission were age, fasting C peptide levels, BMI, duration of T2DM, and the use of insulin preoperatively	RYGB has proven to be an effective and safe option for remission induction in T2DM	5
Zenti et al. [34]. 2015	Observational Study	GB / SG / RYGB	105	T2DM remission was seen in 68.6% of patients. That includes 54/77 for RYGB, 4/11 for GB, and 14/17 for SG patients	RYGB and SG are more effective in achieving remission (as opposed to GB) as they change the upper gastrointestinal tract structure. Factors that increase the probability of remitting are young age, better glycemic control and shorter duration of T2DM	3
Sjöström et al. [35]. 2014	Observational Study	GB / RYGB / LMI / Vertical Banded Gastroplasty	343	At the two-year follow-up period, the T2DM remission rate for surgery patients was 72.3% and 16.4% for the LMI patients. At the 15-year mark, these remission rates decreased to 30.4% for the surgery group and 6.5% for the LMI group	T2DM remission was seen more with bariatric surgery	10
Behbehani et al. [36]. 2014	Observational Study	GB	101	At 24 months post-surgery, complete (HbA1c <42 mmol/mol) and partial (HbA1c 42-48 mmol/mol) remissions of T2DM were achieved in 62.1% and 5.2% of participants, respectively. Mean reduction in BMI of 16.4 kg/m^2^, HbA1c of 23.6 mmol/mol, systolic blood pressure of 12.9 mmHg, and total cholesterol-to-HDL cholesterol ratio of 1.1	Two years postoperatively, RYGB was effective in inducing complete or partial remission in T2DM	2
Dasgupta et al. [37]. 2013	Observational Study	SG	35	At one year postoperatively, HbA1c was 6% in 77.14% of patients without medication. The use of oral hypoglycemic agents reduced from 88.57% to 11.4%	Obese Indian patients with T2DM have shown significant remission rates with SG	1

Discussion

The standard care for T2DM patients has always been lifestyle changes, which include a healthy diet and weight loss, along with regular physical exercise and smoking cessation. If these measures are inadequate in controlling blood glucose levels, pharmacotherapy is introduced, which includes medications like metformin, insulin secretagogues, and eventually, insulin therapy. The problem with insulin therapy and oral hypoglycemic agents is that most of them are known to cause further weight gain, with the exception of dipeptidyl-peptidase-4 (DPP-4) inhibitors and glucagon-like peptide-1 analogues [25]. Bariatric surgery is a modality primarily used for weight reduction in obese patients with comorbidities. Its safety and effectiveness in inducing remission in T2DM should be evaluated. 

Bariatric Surgery Versus Lifestyle and Medical Intervention

Numerous studies have shown that bariatric surgery is superior to LMI alone in achieving remission in T2DM [8,16,21-24,35]. We conducted reviews of six RCTs, as well as an observational study that evaluate the comparison between LMI and bariatric surgery. The referenced articles ranged from the years 2012 until 2016, and sample sizes starting from 32 to 343. The primary endpoint within each of these studies was remission in T2DM [8,16,21-24,35]. 

The complete remission criteria set by the ADA are fasting plasma glucose (FPG) <100 mg/dL and HbA1c <6%, without the use of any hypoglycemic medication for one year [38]. However, many studies use different explanations of what they define as “T2DM remission”. The remission criteria used by Mingrone et al. in RCTs conducted in 2012 and 2015 was the ADA’s partial remission criteria (FPG of <100 mg/dL and HbA1c of <6.5%, with the absence of hypoglycemic agents for one year) as a measure of success rate between bariatric surgery and LMI [8,23]. Additionally, the two RCTs conducted by Courcoulas et al. in 2014 and 2016, designated their complete remission criteria as FPG <100 mg/dL and HbA1c <5.7%, and partial as FPG <125 mg/dL and HbA1c <6.5% [22,24]. Mingrone et al., in 2012, concluded that 75% after RYGB, 95% after BPD, and none of the patients in the LMI group maintained remission at the two-year follow-up [8]. Mingrone et al., in 2015, concluded that patients that underwent RYGB and BPD had remission rates after five years of 37% and 63%, respectively. In contrast, of the 15 medically treated patients, none of them achieved remission at the five-year follow-up period [23]. Furthermore, results from 2014 Courcoulas et al. study showed clear superiority of bariatric surgery with a complete remission rate of 17% with RYGB and 23% with GB, as opposed to 0% in the LMI group at one-year follow-up [24]. In 2015, Courcoulas et al. conducted another RCT that demonstrated results in favour of bariatric surgery again; 40% of patients remitted after RYGB, 29% after GB, and none after LMI. This study also notably concluded that hypoglycemic agents use after surgery was markedly reduced, and even stopped completely at a three-year follow-up [22]. Assessment of the differences in remission criteria between Courcoulas et al. and Mingrone et al. create the case for having a defined and uniform set of conditions by which to check for the primary endpoint for all studies. 

Another study included in our research was an observational study by Sjöström et al. in 2014, which used multiple methods of bariatric surgery, namely, GB, vertical banded gastroplasty, or RYGB for the surgery group, versus LMI for the control group. The study accommodated a sample size of 343 with a follow-up period of 15 years [35]. Alternately, an RCT by Cummings et al., in 2016, compared RYGB exclusively with LMI and included only 32 patients with a follow-up after one year [16]. A major factor within these two studies is that a larger sample size, as exhibited in the Sjöström et al. study, provides a better representation of the T2DM population that ultimately leads to a more statistically accurate study result. Secondly, the larger sample size in the Sjöström et al. study is useful for longer follow-up periods. This is because it ensures that a sufficient sample size exists, despite some individuals naturally being unavailable for follow-up several years after intervention. Thirdly, the longer follow-up period for the Sjöström et al. study allows researchers to detect relapse of T2DM within the sample size and assess for adverse effects of bariatric surgery. The Sjöström et al. study concluded that the control group had a 16.4% remission rate at the two-year follow-up, and 6.5% after 15 years, compared to 72.3% at two years and 30.4% at 15 years for patients who underwent RYGB [35]. On the other hand, the study conducted by Cummings et al. presented that T2DM remission at one year occurred in 60% of patients with RYGB and only in 5.9% with LMI [16]. 

It can be concluded that patients who underwent bariatric surgery achieved T2DM remission in higher numbers compared to patients that were provided just LMI. A significant limitation that we found was that there was no uniform definition of T2DM remission that the studies used. Having a consistent definition is of utmost importance. This mitigates the ability in comparing the different studies, and the efficacy of the methods used for treating T2DM. Even though the ADA has set criteria, many studies were found to be using other definitions. Our suggestion would be to have a consistent, uniform definition of T2DM remission to increase the accuracy of results. We also noticed that many papers did not discuss the possible long-term adverse effects of bariatric surgery. More studies are needed that highlight this topic alongside the benefits of the surgery. A reason for this could be that many studies do not have a follow-up period that would allow a factor as such to be investigated. In this section, the only study that has a 15-year-long follow-up period is Sjöström et al. [35]. Therefore, more studies are needed that follow patients for a long period. 

Comparison of Different Types of Bariatric Surgeries

**Figure 2:**
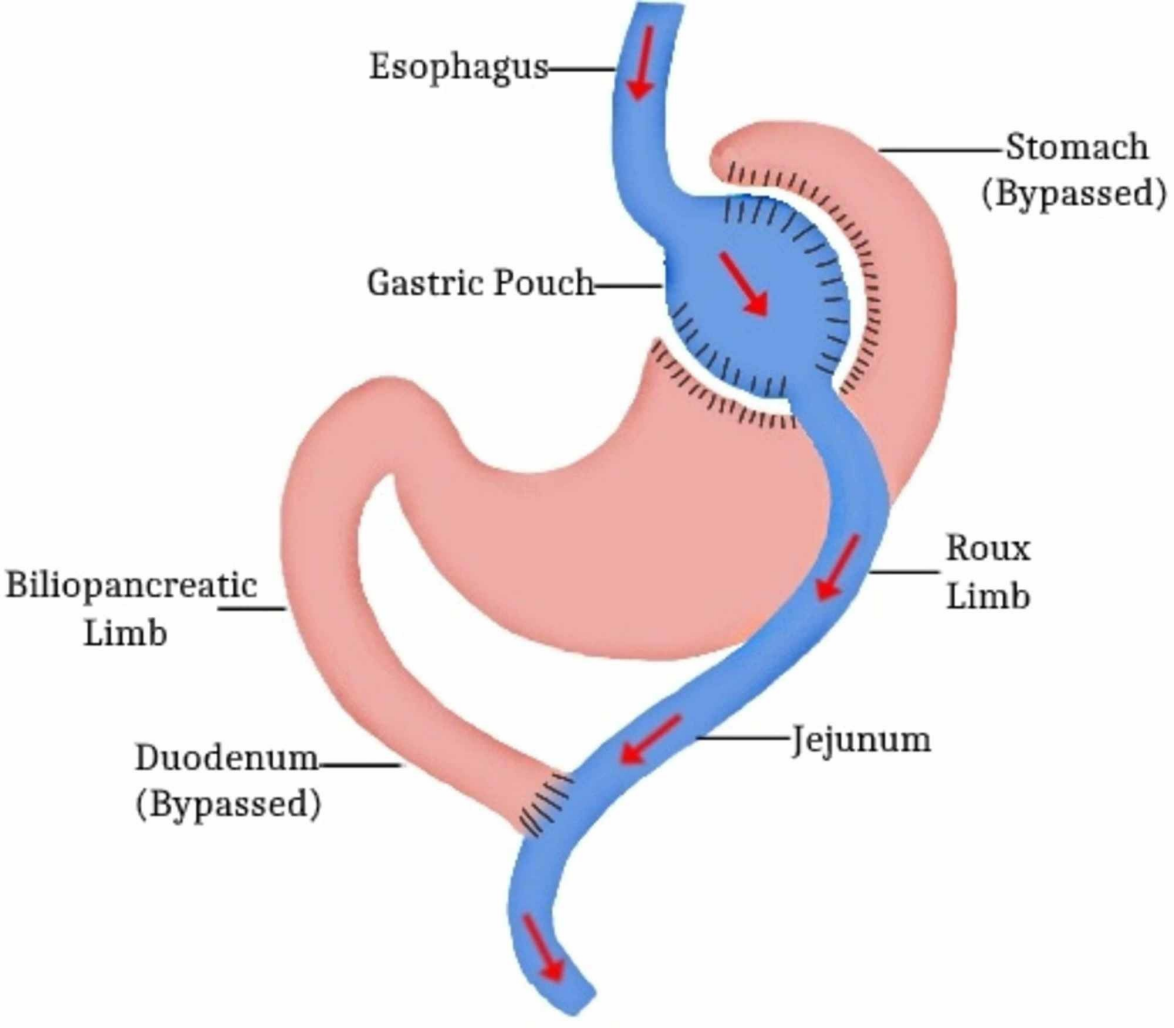
Roux-en-Y Gastric Bypass

There are many different types of bariatric surgeries. These include RYGB, SG, GB, BPD, and One-Anastomosis Gastric Bypass (OAGB). RYGB and SG have been greatly studied for their efficacy in remitting T2DM (Figure 2).

Several observational studies focused on just a single type of bariatric surgery and provided comprehensive findings for its efficacy. Studies conducted by Dasgupta et al. and Nedelcu et al. focused entirely on SG. The conclusion achieved within both of these studies was consistent in that sleeve gastrectomy resulted in T2DM remission in over 50% of patients [7,37]. Studies conducted by Behbehani et al., Adams et al., and Madsen et al. emphasized their research on the efficacy of RYGB alone on remitting T2DM [26,29,36]. The conclusion arrived was consistent across the board as RYGB resulted in remission at each follow-up period. 

The purpose of our research is to identify which bariatric surgery method is more effective in showing remission in T2DM patients, as well as maintaining that remission. The studies focusing on RYGB had drastically larger sample sizes with 101, 88, and 1,111 for Behbehani et al., Adams et al., and Madsen et al., respectively, compared to their counterparts assessing SG like the Dasgupta et al. study that had 35 patients and the Nedelcu et al. study that had 46 [7,26,29,36,37]. The larger sample sizes for the RYGB studies make their results more applicable to T2DM patients [26,29,36]. Each of the SG studies had a follow-up at the one-year mark, with only Nedelcu et al. performing a secondary check on the patients at five years post-surgery [7,37]. The different follow-up periods for the RYGB studies were two years, five years, and 12 years. Due to the longer follow-up periods, especially with the Adams et al. study, and the large sample size with Madsen et al. study, it can be concluded that RYGB proves to be a better modality [26,29,36]. 

The Adams et al. study gives an insightful look into the realities of long follow-up periods and their effect on T2DM remission rates. It displayed this with 75% remission at two years, 62% at six years, and consistently declining to 51% at 12 years [29]. Similarly, the Madsen et al. study had 74% remission that patients showed at the one-year follow-up, however, 27% of those patients relapsed after five years [26]. This seems to be consistent with other studies where patients are assessed at different intervals postoperatively and the remission rates decrease. 

A 2019 RCT serves as evidence of which method is more reliable and effective. This RCT was conducted by Hofsø et al. in Norway and assessed remission at one year postoperatively. They compared SG and RYGB, and their impact on T2DM remission rates. The study results proved that RYGB is more effective than SG. The reasons why results from the Hofsø et al. study are accurate and more representative is because it is an RCT, which is inherently higher on the pyramid of evidence and is a more recent study with a good sample size. Additionally, it is one of the few studies that incorporated a triple-blind protocol, creating an environment ideal for mitigating bias within research [15]. 

An observational study from 2015 by Yska et al. compared three different surgical methods. This study included RYGB and SG, and a third procedure, GB. By assessing the overall effect of each surgery method on the test group, Yska et al. concluded that two years after surgery, RYGB had the greatest decrease in HbA1c and blood glucose, followed by SG and then GB [32]. Another similar observational study by Wazir et al. was conducted in 2019. This study also included a comprehensive view of GB, SG, and RYGB. This study resembled the 2015 Yska et al. study as it concluded with RYGB being superior to the other methods with greater remission rates at the two-year mark [14]. 

Similarly, the observational study by Purnell et al. in 2016 compared the effects of RYGB and GB. After a three-year follow-up period, the study concluded that 68.7% of patients in the RYGB group and 30.2% in the GB group were in T2DM remission. Therefore, patients within the RYGB group achieved higher remission rates. An interesting finding was that during the annual follow-ups, the number of RYGB patients in remission decreased as the years progressed, from 71% in year one to 68.7% in year three, compared to GB patients who slightly rose from 29.9% in year one to 30.2% in year three. This infers that regardless of the initial remission rate, patients can maintain sustainable remission after GB in the postoperative years [31]. Further studies with longer follow-up periods are needed to assess if this is true on a long-term scale. 

BPD is another type of bariatric procedure, which was included in the observational study by Fernández-Soto et al. conducted in 2017. Alongside BPD, they assessed the efficacy of SG and RYGB. They had a sample size of 49 patients. Contrary to other studies, they concluded that there was no difference in T2DM remission rates after the three procedures, and a substantial 80% of patients had remitted at a one-year follow-up. They mention that this finding could be because only morbidly obese (BMI >50.2kg/m^2^) patients were included in their study, as opposed to other studies (which include patients with lower BMIs) that show a clear superiority of RYGB over other procedures [28]. This finding from the Fernández-Soto et al. study can be compared with results from Behbehani et al., where participants had a BMI >50.3kg/m^2^ and RYGB was still the dominant procedure for attaining remission. There was also a larger sample size and a longer follow-up period in the Behbehani et al. study [36]. However, it should be noted that the research by Fernández-Soto et al. is relatively more recent. 

A recent 2019 study conducted by Ruiz-Tovar et al., including 546 patients, investigated the differences in remission attained through SG, RYGB, and OAGB. The findings from this study differed from other research, as they concluded that OAGB was superior to RYGB and SG in remitting T2DM at a five-year follow-up. There was also a greater decrease in BMI after OAGB, compared to RYGB and SG. Furthermore, T2DM remission in RYGB was not significantly higher than with SG, a unique discovery not found in other studies. Due to its study design as an RCT, the Ruiz-Tovar et al. study had more credibility, as patients were randomly assigned to intervention type [20]. 

Overall, RYGB conclusively proves more effective for patients with BMI >35kg/m^2^. A limitation found while searching for data was the shortage of RCTs specifically catered towards the comparison of different bariatric surgical methods, especially those that incorporate a double/triple-blind protocol. Additionally, there is a shortage of RCTs targeting bariatric surgery for morbidly obese T2DM patients with BMI >50. The OAGB procedure seems to be a promising alternative to treating T2DM, however, more RCTs should focus on this particular modality to check for remission rates. In addition, larger sample sizes and longer follow-up periods are needed. 

Preoperative Predictive Factors

To ensure that bariatric surgery is performed only on those patients that could see possible remission, we need to research different factors resulting in a good post-op prognosis. These factors affect not only the efficacy of surgery, but also the long-term implications. We researched different observational studies to determine these clinical factors. 

A study conducted by Zenti et al., with 105 patients, showed that the best outcome seen after bariatric surgery was having a <two-year-long diabetes duration, exhibiting good glycemic control (HbA1c <7%), and age of <45 years [34]. Comparatively, we reviewed the results of the Bhasker et al. study, with 106 patients, and noted that the different pre-op predictors of remission were age <60 years, BMI >40 kg/m^2^, fasting C-peptide levels >3, duration of T2DM <five years, and no pre-op insulin use [33]. Both of these studies are observational, both were conducted in 2015, and have similar sample sizes. However, the study by Bhasker et al. had a follow-up after five years, compared to Zenti et al., which was after three years. As a result, the Bhasker et al. study proves to be relatively more reliable. In reference to no prior insulin use, the Ghio et al. study conducted in 2017 in Spain deserves to be mentioned. They included patients that were treated with insulin before bariatric surgery. The initial remission rates, at a median of 11.7 months, amounted to only 27%, of which 65% relapsed at a median of 26.7 months. Therefore, due to the large number of individuals who relapsed post-surgery, it can be concluded that pre-op insulin use is an indicator of a worse prognosis after bariatric surgery [30]. 

Alongside the many different factors that play a role in inducing remission after bariatric surgery, one noteworthy factor is the level of an inflammatory marker, osteopontin. This was researched by a study in 2019 by Carbone et al. They included 41 patients in their study and arrived at the conclusion that at the three-year follow-up, high levels of osteopontin correlated with greater T2DM remission. They also discovered that baseline osteopontin levels predicted remission independent of weight loss, baseline BMI and duration of diabetes. Serum osteopontin levels of >46.57 mg/mL predicts possible remission [27]. 

Honarmand et al. focused their research on the DiaRem score, which is a scoring system to predict T2DM remission post-bariatric surgery. The baseline variables that are assessed in this scoring system are the age of the individual, HbA1c level, insulin therapy, and use of oral antidiabetic medication like insulin sensitizers or sulfonylureas. Nine hundred patients were included in this study to check for the efficacy of the DiaRem score. The findings showed that at one-year follow-up after RYGB, 37% of individuals had remitted, and the clinical factors within the DiaRem score were associated with this remission. A helpful DiaRem cutoff score of <5 (71.8% sensitivity, 71.3% specificity) was established by this study, and they concluded that this scoring system had a moderate predictive value at best [1]. 

In light of the evidence available from these studies, it is safe to conclude that age of <60 years, duration of T2DM of <5 years, BMI >40 kg/m^2^, HbA1c <7%, fasting C-peptide levels of >3, osteopontin level >46.57 mg/mL, no preoperative insulin treatment, and a DiaRem score of <5 indicate good prognosis after bariatric surgery. A limitation that was felt while searching for predictive factors was that not many studies were RCTs, especially the ones that specifically focus on preoperative clinical factors. More double- and triple-blind RCTs should focus on this crucial factor. Another suggestion that we would like to include is that scoring systems like the DiaRem should be researched more in RCTs and used widely by physicians in choosing the right individuals for the intervention. In addition, the correlation of levels of osteopontin and T2DM remission should be studied more using a large sample size with a prolonged follow-up period to come to a definitive conclusion about this correlation. In addition, more inflammatory markers like this one should be researched as possible indicators of remission in RCTs. 

Limitations

One of the limitations of our study was a scarcity of double/triple-blind RCTs. Within the selected RCTs, the patients were randomly assigned but were not blind to the intervention. In addition, many studies did not use a consistent definition for T2DM remission. We also only chose English language papers, which hindered our ability to find quality literature in other languages. Furthermore, our research did not include Type 1 Diabetes Mellitus patients, individuals with mild obesity, or pre-diabetes. As a result, we were unable to acquire a complete understanding of the effects of bariatric surgery in those populations, and how that compares with effects on T2DM. 

## Conclusions

This systematic review focused on comparing the effects of different types of bariatric surgeries with LMI on T2DM remission, along with preoperative factors that help achieve this remission. Based on the review, it appears that bariatric surgery is superior to LMI in inducing remission in T2DM patients, and especially the RYGB modality. The perfect candidate would be an individual aged <60 years with a BMI >40kg/m^2^, having a <5-year long T2DM duration, an HbA1c level of <7%, a fasting C-peptide level of >3, and no prior insulin treatment. Along with these factors, having an osteopontin level of >46.57 mg/mL and a DiaRem score of <5 is a great indicator of remission postoperatively. Given the high prevalence of T2DM and the associated complications, our systematic review will be very useful for physicians in selecting appropriate individuals for surgery, who will truly benefit from the intervention. More double- or triple-blind RCTs with a uniform definition of T2DM remission, large sample sizes, and prolonged follow-up periods (to assess for long term adverse effects and relapses in remission) are needed. Additional research is required in assessing remission in those with BMI >50 kg/m^2^, the OAGB modality, scoring systems like the DiaRem, and the role of osteopontin along with other possible inflammatory markers as predictors.
